# Perioperative hypoalbuminemia is a risk factor for wound complications following posterior lumbar interbody fusion

**DOI:** 10.1186/s13018-020-02051-4

**Published:** 2020-11-17

**Authors:** Zhongyuan He, Kai Zhou, Ke Tang, Zhengxue Quan, Shaoyu Liu, Bao Su

**Affiliations:** 1 Department of Orthopedics, The Seventh Affiliated Hospital, Sun Yat-sen University, Guangming District, Shenzhen, 518000 China; 2 Department of Orthopedics, The First Affiliated Hospital of Chongqing Medical University, Yuanjiagang, Yuzhong District, Chongqing, 400016 China

**Keywords:** Hypoalbuminemia, Malnutrition, Risk factors, Single-segment lumbar fusion, Surgical wound dehiscence, Retrospective studies

## Abstract

**Background:**

Although serum albumin levels are increasingly used as an indicator of nutritional status in the clinic, the relationship between perioperative hypoalbuminemia and wound complications after posterior lumbar interbody fusion in the treatment of lumbar degenerative disease remains ambiguous. The aim of this study was to evaluate perioperative serum albumin in relation to postoperative wound complications after posterior lumbar interbody fusion in the treatment of single-segment lumbar degenerative disease.

**Material and methods:**

We reviewed patients who underwent single-segment posterior lumbar interbody fusion surgery from December 2014 to April 2017 in the Department of Orthopedics at the First Affiliated Hospital of Chongqing Medical University. Perioperative (preoperative and early postoperative) serum albumin levels were assessed in all patients and were used to quantify nutritional status. We divided the patients into a surgical wound dehiscence (SWD) group and a normal wound healing group and into a surgical site infection (SSI) group and a non-SSI group. To evaluate the relationship between perioperative serum albumin level and postoperative wound complications, we conducted univariate and multiple logistic regression analyses.

**Results:**

A total of 554 patients were enrolled in the study. The univariate and multiple logistic regression analysis of these differences showed that preoperative serum albumin < 3.5 g/dl (*P* = 0.001) and postoperative serum albumin < 3.0 g/dl (*P* = 0.001) were significantly correlated to SWD. There were also significant differences between the SSI groups in terms of preoperative serum albumin < 3.5 g/dl (*P* = 0.001) and chronic steroid use (*P* = 0.003). Additionally, the increased hospitalization costs (*P* < 0.001) and length of hospitalization (*P* < 0.001) were statistically significant for patients with perioperative hypoalbuminemia.

**Conclusions:**

For patients who underwent single-segment posterior lumbar interbody fusion surgery, we need to pay more attention to perioperative hypoalbuminemia and chronic steroid use, which are more likely to be associated with increased wound complications, hospitalization costs, and length of hospitalization after surgery. Adequate assessment and management of these risk factors will help reduce wound complications and hospital stays for surgical patients and will save medical costs.

## Background

Nutritional status is a key factor in patient prognosis in various medical contexts [1–4]. An estimated 4.3% of community-dwelling adults suffer from malnutrition, and the prevalence of malnutrition among medical inpatients ranges from 20 to 45% [5–11]. Malnutrition can be identified in various ways, including serological marker evaluation, anthropometric measurements, and nutrition scoring tools. Among the numerous methods to define malnutrition, the one most frequently used is the serum albumin level. Albumin less than 3.5 g/dL is recognized as hypoalbuminemia (malnourished) [12–14].

The perioperative malnutrition of surgical patients is related to various postoperative adverse outcomes, including infection, acute kidney injury (AKI), and mortality. Wound complications are the most common problems for surgeons. Understanding the impact of perioperative malnutrition on postoperative wound complications and outcomes is of great significance.

Lumbar fusion is one of the most common spinal procedures. Although this is a relatively safe operation with a high success rate, there is still a risk of postoperative complications and a need for revision surgery. The common wound complications are wound infection, wound dehiscence, wound hematoma, and wound hernia. The incidence of complications after spinal fusion ranges from 1 to 20% [15, 16]. Wound complications increase patient suffering, hospital length of stay, readmission rate, medical expenses, and mortality and place a heavy burden on patients’ families and social health systems. Although progress has been made in the effective prevention of infection through antimicrobials, strengthening wound area management, strengthening operating room, instrument disinfection control, strict suturing during surgery, postoperative drainage, and other methods reduce the possibility of complications. It is still difficult to avoid wound complications in some spinal patients after surgery.

In recent years, with the gradual increase in patients with lumbar degenerative diseases, we found that perioperative malnutrition also increased year by year. Perioperative malnutrition may be linked to age, poor eating, pain, long-term bed rest, and other factors. Obese patients who are overweight may still be malnourished [17–19]. Despite the growing body of literature on the effects of hypoalbuminemia on postoperative outcomes in other surgical fields, few studies to date have investigated the relationship between perioperative hypoalbuminemia and postoperative wound complications in patients following degenerative lumbar spine surgery. The aim of the study was to explore whether the perioperative albumin level can be used as a risk indicator of postoperative wound complications after one-level posterior lumbar interbody fusion (PLIF).

## Material and methods

In The First Affiliated Hospital of Chongqing Medical University, 787 folders of all consecutive patients operated by PLIF were reviewed between December 2014 and April 2017. The study protocol was conformed to the ethical guidelines of the Declaration of Helsinki and the Ethics Committee of The First Affiliated Hospital of Chongqing Medical University, Chongqing, China, and all patients provided informed consent concerning the use of their medical records.

The inclusion criteria were (1) patients aged of 18 or above; (2) patients with a diagnosis of primary single-segment lumbar degenerative disease, lumbar disc herniation, lumbar spinal stenosis, or spondylolisthesis; (3) patients undergoing PLIF surgery; and (4) patients followed up for a minimum of 1 year with complete data.

The exclusion criteria were the following: (1) patients with multisegment lumbar degenerative disease, (2) patients with previous lumbar surgery, and (3) patients with incomplete laboratory data.

All operations were performed in standard vertical stratospheric operating rooms. We performed antibiotic prophylaxis 30 min before the beginning of the surgery and prolonged to the first 72 h postoperatively. All operations were performed by the members of the same medical team (from the authors). Patients were encouraged to wear braces after surgery.

The main preoperative baseline variables included albumin level; the secondary preoperative baseline variables included age, body mass index (BMI), sex, diabetes, tobacco use, and chronic steroid use. Considering that early bed rest in patients with lumbar spondylolisthesis may lead to postoperative wound complications, we also included the preoperative diagnosis in the evaluation index.

We assessed two wound-related complications: SWD and SSI. According to the Centers for Disease Control (CDC) and Prevention criteria [20], superficial SSI was defined as follows: (a) purulent drainage from the superficial incision; (b) organism(s) identified from an aseptically obtained specimen from the superficial incision or subcutaneous tissue by a culture- or non-culture-based microbiologic testing method which is performed for purposes of clinical diagnosis or treatment; (c) the wound had at least one of the following signs of symptoms: localized pain or tenderness, localized swelling, erythema, or heat; and (d) diagnosis of superficial incisional surgical site infection by the surgeon, attending doctor, or other designee.

For deep SSI, a surgical wound was with at least one of the following: (a) purulent drainage from the deep incision; (b) organism(s) identified from the deep soft tissues of the incision by a culture- or non-culture-based microbiologic testing method which is performed for purposes of clinical diagnosis or treatment, and when the patient has at least one of the following signs and symptoms: fever (> 38 °C), localized pain, or tenderness; and (c) an abscess or other evidence of infection involving the deep incision that is detected on gross anatomical or histopathologic exam, or imaging test. Patients meeting the above criteria were included in the SSI. During the hospital stay and follow-up, the surgeons remained vigilant for any signs of wound complications (drainage, atherosclerosis, skin necrosis, and dehiscence) in these patients. A surgical site infection occurring within 1 year after surgery was also considered as an infectious complication.

Preoperative serum albumin and postoperative short-term albumin levels within 7 days of surgery were collected. Operative variables included operative time and intraoperative blood loss. Postoperative variables included postoperative drainage amount and length of hospital stay. Overall hospital costs in Chinese Yuan (CNY) were also reported. Albumin serum level less than 3.5 g/dL was recognized as hypoalbuminemia, and when the level was less than 3.0 g/dL, an intravenous albumin supplementation was required [9–11].

### Statistical analysis

Statistical analyses were performed using SPSS 24.0 for Windows. Data are shown as the mean ± standard deviation (SD) and median (interquartile range, IQR). Logistic regression analysis was conducted to analyze the risk factors for SWD and SSI. Univariate logistic regression found factors with *p* < 0.05 that were added to multivariate logistic regression analysis. Odds ratios (ORs) and 95% confidence intervals (CIs) were also determined. A *p* value of less than 0.05 was considered as statistically significant. The Student *t* test and Mann-Whitney *U* test were used for comparing demographic data between the two groups. All *p* values were two-sided.

## Results

A total of 787 patients were eligible, but only 554 patients were included in this study. There were 317 females and 237 males. The mean age of the patients was 56.9 years (range 22–85). The incidence of SWD after lumbar spine fusion was 12.45% (69/554), and the incidence of SSI was 3.43% (19/554). Microorganisms were isolated from all 19 patients with SSI by open debridement, ultrasound-guided biopsy, superficial exudate, and drainage culture. There were 13 (2.4%) patients with malnutrition before surgery (preoperative albumin < 3.5 g/dL), 314 (56.4%) patients with albumin < 3.5 g/dL within 7 days after surgery, and 114 (20.6%) patients with albumin < 3.0 g/dL within 7 days after surgery (Tables 1 and 2).

**Table 1:** Patients’ demographic characteristics for SWD

Variable	Total (*N* = 554)	Surgical wound dehiscence (*n* = 69)	Normal wound healing (*n* = 485)	*P* value
**Preoperative baseline variables**				
Age at surgery, mean (SD)	56.9 (13.4)	60.8 (14.2)	56.4 (13.2)	0.950
BMI, mean (SD)	24.1 (3.1)	24.2 (3.8)	24.1 (3.0)	0.731
Male	237 (43)	30 (43.5)	207 (42.7)	0.076
Diabetes	60 (10.8)	9 (13.0)	51 (10.5)	0.965
Smoker	144 (26.0)	22 (31.9)	120 (25.2)	0.573
Chronic steroid use	11 (1.99)	5 (7.25)	6 (1.24)	< 0.001
Albumin level < 3.5 g/dl	13 (2.35)	6 (8.70)	7 (1.44)	0.001
Diagnose				
Herniation	270 (48.7)	33 (47.8)	237 (48.9)	-
Stenosis	86 (15.5)	12 (17.4)	74 (15.3)	0.053
Spondylolisthesis	198 (35.7)	24 (34.8)	174 (35.8)	0.397
**Operative variables**				
Operative time, mean (SD), min	160.8 (43.9)	154.4 (48.5)	161.8 (43.2)	0.593
Bleeding volume, mean (SD) mL	318.5 (90.8)	284.1 (30.4)	311.4 (89.6)	0.662
**Postoperative variables**				
Albumin level < 3.5 g/dl	314 (56.7)	56 (81.2)	258 (53.2)	0.023
Albumin level < 3.0 g/dl	110 (19.9)	36 (52.2)	74 (15.3)	0.001
Postoperative drainage median, ml, (IQR)	223.5 (151.0–309.0)	205.0 (120.0–300.0)	231.0 (154.5–310.5)	0.003
Length of stay, day, median (SD)	11.0 (9.0–14.0)	17.0 (13.0–22.5)	11.0 (9.0–13.0)	< 0.001
Hospitalization expenses mean, thousand CNY (SD)	25.6 (8.7)	35.0 (17.5)	24.2 (5.5)	< 0.001

*SWD* Surgical wound dehiscence, *BMI* Indicates body mass index, *SD* Standard deviation, *CNY* Chinese Yuan, *IQR* Interquartile range

*Statistically significant (*P* < 0.05)

**Table 2:** Patients’ demographic characteristics for SSI

Variable	Total (*N* = 554)	SSI (*n* = 19)	No-SSI (*n* = 535)	*P* value
**Preoperative baseline variables**				
Age at surgery, mean (SD)	56.9 (13.4)	56.7 (16.0)	56.9 (13.3)	0.010
BMI, mean (SD)	24.1 (3.1)	23.9 (3.9)	24.1 (3.1)	0.750
Male	237 (43)	12 (63.2)	225 (42.1)	0.900
Diabetes	60 (10.8)	2 (10.5)	58 (10.8)	0.300
Smoker	144 (26.0)	6 (31.6)	138 (25.8)	0.235
Chronic steroid use	11 (1.99)	5 (26.3)	6 (1.1)	0.003
Albumin level < 3.5 g/dl	13 (2.35)	3 (15.8)	10 (1.9)	0.001
Diagnose				
Herniation	270 (48.7)	6 (31.6)	264 (49.3)	-
Stenosis	86 (15.5)	6 (31.6)	80 (15.0)	0.180
Spondylolisthesis	198 (35.7)	7 (36.8)	191 (35.7)	0.001
**Operative variables**				
Operative time, mean (SD), min	160.8 (43.9)	155.5 (48.2)	161.0 (43.8)	0.178
Bleeding volume, mean (SD) mL	318.5 (90.8)	321.1 (105.7)	311.9 (89.2)	0.537
**Postoperative variables**				
Albumin level < 3.5 g/dl	314 (56.7)	16 (84.2)	298 (55.7)	< 0.001
Albumin level < 3.0 g/dl	110 (19.9)	10 (52.6)	100 (18.7)	< 0.001
Postoperative drainage median, ml, (IQR)	223.5 (151.0–309.0)	148 (93.0-188.0)	231 (154.0–312.0)	0.116
Length of stay, day, median, thousand CNY (SD)	11.0 (9.0–14.0)	22.0 (14.0–32.0)	11.0 (9.0–14.0)	< 0.001
Hospitalization expenses mean (SD)	25.6 (8.7)	43.9 (21.3)	24.9 (7.2)	< 0.001

*SSI* Surgical site infection, *BMI* Indicates body mass index, *SD* Standard deviation, *CNY* Chinese Yuan, *IQR* Interquartile range

*Statistically significant (*P* < 0.05)

Chronic steroid use (*P* < 0.001), preoperative albumin level < 3.5 g/dl (*P* = 0.001), postoperative albumin level < 3.5 g/dl (*P* = 0.023), postoperative albumin level < 3.0 g/dl (*P* = 0.001), and postoperative drainage (*P* = 0.003) were significantly correlated with the incidence of SWD.

Age (*P* = 0.010), chronic steroid use (*P* = 0.003), preoperative albumin level < 3.5 g/dl (*P* = 0.001), spondylolisthesis diagnosis (*P* = 0.001), postoperative albumin level < 3.5 g/dl (*P* < 0.001), postoperative albumin level < 3.0 g/dl (*P* < 0.001), length of stay (*P* < 0.001), and hospitalization expenses (*P* < 0.001) were significantly correlated with the incidence of SSI.

**Fig. 1:**
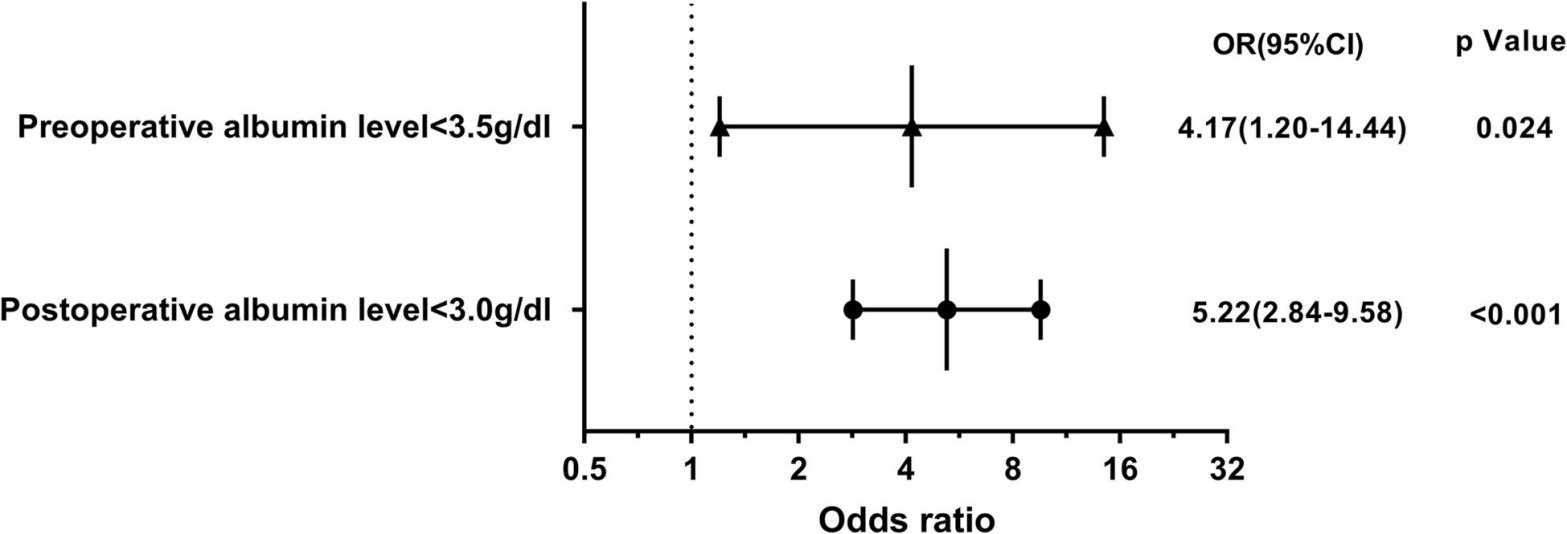
Forest plot of multivariate logistic regression for SWD. Preoperative albumin level < 3.5 g/dl and postoperative albumin level < 3.0 g/dl were statistically significant risk factors for SSI. OR, odds ratio

**Fig. 2:**
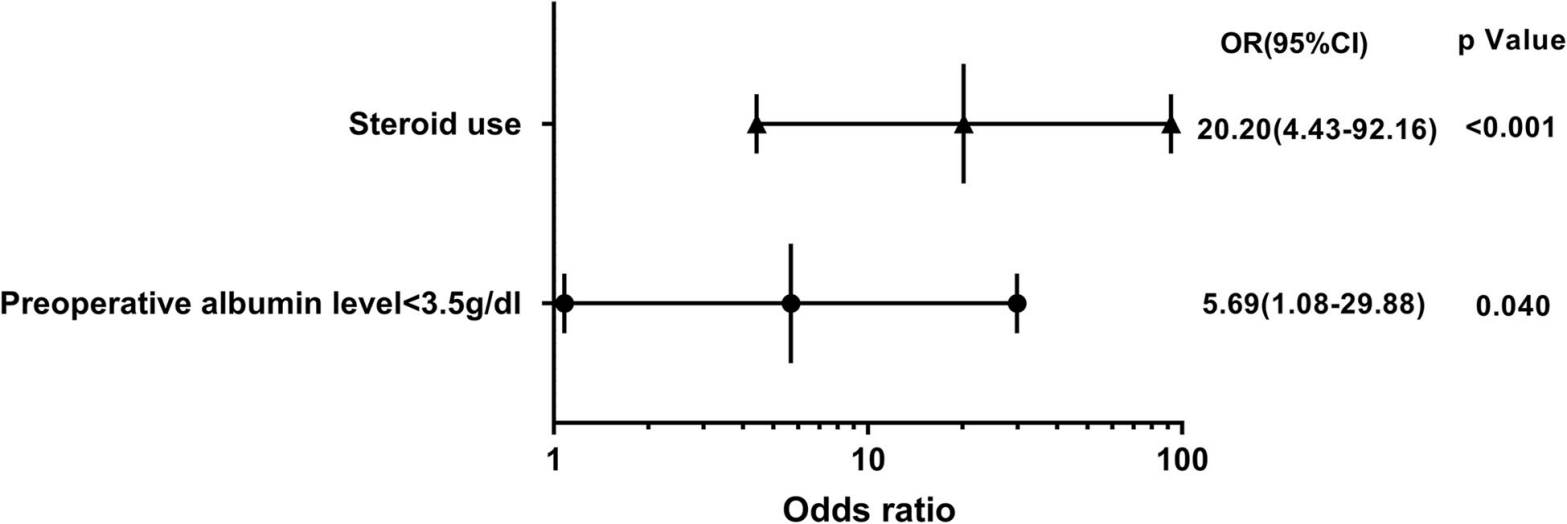
Forest plot of multivariate logistic regression for SSI. Preoperative albumin level < 3.5 g/dl and chronic steroid use were statistically significant risk factors for SSI. OR, odds ratio

Multivariate logistic regression showed that preoperative albumin level < 3.5 g/dl (*p* = 0.024, OR = 4.16, 95% CI 1.203–14.44) and postoperative albumin level < 3.0 g/dl (*p* < 0.001, OR = 5.22, 95% CI 2.84–9.58) were risk factors for SWD (Table 3, Fig. 1). Multivariate logistic regression also found that preoperative albumin level < 3.5 g/dl (*p* = 0.040, OR = 5.69, 95%CI 1.08–29.88) and chronic steroid use (*p* < 0.001, OR = 20.20, 95%CI 4.43–92.16) were statistically significant risk factors for SSI (Table 4, Fig. 2).

**Table 3:** Multivariate logistic regression analysis (SWD)

Variable	*B*	SD	OR	95% CI	*P* value
Preoperative					
Albumin level < 3.5 g/dl	1.427	0.634	4.168	1.203–14.442	0.024
Postoperative					
Albumin level < 3.5 g/dl	0.540	0.364	1.716	0.841–3.503	0.138
Postoperative					
Albumin level < 3.0 g/dl	1.652	0.310	5.219	2.843–9.580	< 0.001
Age	0.016	0.011	1.017	0.995–1.039	0.133
Chronic steroid use	1.378	0.722	3.967	0.965–16.317	0.056

*SWD* Surgical wound dehiscence, *B* a constant which indicates regression coefficient and intercept, *SD* Standard deviation, *OR* Odds ratio, *CI* Confidence interval

*Statistically significant (*P* < 0.05)

**Table 4:** Multivariate logistic regression analysis (SSI)

Variable	*B*	SD	OR	95% CI	*P* value
Preoperative					
Albumin level < 3.5 g/dl	1.738	0.846	5.687	1.082–29.880	0.040
Postoperative					
Albumin level < 3.5 g/dl	0.886	0.717	2.425	0.595–9.889	0.217
Postoperative					
Albumin level < 3.0 g/dl	0.695	0.576	2.003	0.647–6.196	0.228
Drainage	0.008	0.003	0.993	0.986–0.999	0.023
Chronic steroid use	3.006	0.774	20.203	4.429–92.155	< 0.001

*SSI* Surgical site infection, *B* A constant which indicates regression coefficient and intercept, *SD* Standard deviation, *OR* Odds ratio, *CI* Confidence interval

*Statistically significant (*P* < 0.05)

The average hospital stay length was 11 (9–14) days overall; it was 17 (13–22.5) days in the SWD group and 11 (9–13) days in the normal wound healing group. Length of hospital stay in the SSI group was 22 (14–32) days and in the non-SSI group was 11 (9–14) days, with a significant difference (*p* < 0.001). The average patient expenditure during hospitalization was 25.6 (± 8.7) thousand CNY (US$ 3583.2 ± 1217.7); overall, it was 35.0 (±17.5) thousand CNY (US$ 4898.9 ± 2449.5) in the SWD group, and it was 24.2 (± 5.5) thousand CNY (US$ 3387.2 ± 769.8) in the normal wound healing group (*P* < 0.001). The average patient expenditure during hospitalization in the SSI group was 43.9 (± 21.3) thousand CNY (US$ 6144.6 ± 2981.3), and in the non-SSI group, it was 24.9 (± 7.2) thousand CNY (US$ 3485.2 ± 1007) (*P* < 0.001).

## Discussion

The current study confirmed that perioperative hypoalbuminemia was an important risk factor for wound complications following single-segment PLIF surgery. Our study further confirmed that perioperative hypoalbuminemia is an important risk factor for SWD and SSI.

Previous studies have reported that various markers of preoperative malnutrition are associated with surgical site infection following various types of surgery [21–27]. Cross et al. pointed out that superficial and deep SSI after orthopedic spinal surgery were associated with several markers of malnutrition, such as serological laboratory values, diabetes mellitus, hyperglycemia, and obesity [28]. Bohl et al. investigated the relationship between preoperative hypoalbuminemia and complications within 30 days after total joint replacement. Compared with patients with normal albumin concentrations, patients with hypoalbuminemia had a higher risk of surgical site infection, pneumonia, prolonged hospital stay, and readmission [29]. Similarly, they retrospectively reviewed data prospectively collected by the American College of Surgeons National Surgical Quality Improvement Program to investigate the relationship between preoperative hypoalbuminemia and complications after 30 days of posterior lumbar fusion. They pointed out that malnutrition was an independent risk factor for infection and wound complications after posterior lumbar fusion, and it was also associated with increased length of stay and readmission [24].

Our data showed that preoperative low serum albumin (< 3.5 g/dL) was significantly associated with an increased risk of postoperative SWD (*P* = 0.024) and SSI (*P* = 0.040), which is consistent with other related reports. We combined the results of these data with previous literature and concluded that preoperative serum albumin concentration can not only be used to measure nutritional status, but also is more closely related to poor wound healing and pathological inflammation. It is a reliable indicator for evaluating postoperative complications

In addition, early postoperative hypoalbuminemia has also been reported as a risk factor for serious postoperative complications [30–33]. Lee et al. investigated 337 patients with major oropharyngeal squamous cell carcinoma who underwent clean and contaminated surgery and monitored serum albumin, glucose, and hemoglobin levels during the perioperative period. The results showed that early postoperative hypoalbuminemia < 2.5 g/dl was an independent risk factor for SSI in patients who underwent oral cancer surgery [34]. Bohl et al. also reported that malnutrition increased the risk of periprosthetic joint infection following total joint arthroplasty [35].

Low postoperative serum albumin (< 3.0 g/dL) was significantly associated with an increased risk of postoperative SWD. However, low postoperative albumin is affected by many factors. Ge et al. noted that the stress response, perioperative fluid overload, hemodilution, albumin redistribution, a breakdown of metabolism, and other comprehensive factors cause postoperative albumin decline [36]. Despite this, we still need to be wary of hypoalbuminemia with albumin levels < 3.0 g/dl after surgery. Considering low hypoalbuminemia mechanisms, monitoring the albumin serum level and giving albumin supplementation in case of low level appeared to be an adequate strategy to decrease the risk.

Chronic steroid users (steroid usage for more than 10 days preoperatively) have reportedly increased their risk of infection by two- to three-fold after surgery. Singla et al. reported chronic steroid usage to be a significant risk factor for SSI in their database analysis of 360,005 patients over 65 years of age [37].

Univariate logistic regression showed that poor drainage after surgery could lead to SWD. This may be related to the poor placement of the drainage tube and blockage of the drainage tube. Irregular drainage after surgery is prone to deep congestion and hematoma, and then, SWD and also increased risk of SSI. Although the *p* value of postoperative drainage (*P* = 0.003) was less than 0.05, OR value was close to 1 infinitely, which may be related to the small sample size of SSI group. Expanding the sample size would be helpful for further research. Increased risk of SSI in patients with spondylolisthesis could be explained by prolonged bed rest after PLIF surgery, considering that short-term (7 days) could be beneficial to the stability of the spine after reduction to prevent loosening or displacement of the internal fixation. Age is also a risk factor for SSI, with the increase of age, the probability of SSI after the operation is higher. The finding may be the result of malnourished conditions due to poorer nutritional intake. However, this should be further evaluated in future prospective experiments with increased sample sizes.

When malnutrition is detected, timely nutritional supplementation is beneficial to patients’ postoperative recovery. Oral nutritional supplements have been shown to be effective in improving nutrient intake, and they can also be given intravenously. Avenell et al. proved that oral non-protein energy, protein, vitamin, and mineral supplements can prevent complications after hip fracture in elderly patients [38].

The strengths of this study include the use of the same surgical procedure (PLIF) for lumbar fusion and internal fixation. Only single-segment fusion patients were included to reduce the impact of surgical procedures on the results of the study. In future research, it will be important to further explore, elucidate, and establish potential links between hypoalbuminemia and adverse surgical wound outcomes after spinal fusion surgery.

The study was limited by the inherent problems of retrospective studies. First, as a retrospective, single institution study, all data on patient characteristics, laboratory test results, medical interventions directly related to abnormal laboratory values, and patient clinical symptoms were dependent on the inherent limitations of the files in the electronic medical record system. Second, the sample size of patients with preoperative hypoalbuminemia (13 cases) was relatively small, and a larger sample size may have been helpful for the statistical analysis. Although the serum albumin value is a valuable tool for assessing nutritional status, it is affected by many perioperative factors, so it cannot be a comprehensive assessment of the nutritional status of patients. We still need to integrate the general situation of patients and other serological indicators to guide clinical treatment.

## Conclusions

The current study showed that lower preoperative (< 3.5 g/dl) and postoperative (< 3.0 g/dl) serum albumin values were associated with SWD, and lower preoperative (< 3.5 g/dl) serum albumin levels and chronic steroid use were associated with SSI after posterior lumbar interbody fusion in the treatment of single-segment lumbar degenerative disease. More attention should be paid to the nutritional status of patients to ensure they are supplemented in a timely manner and to reduce hospitalization time and costs.

## Data Availability

The datasets used and/or analyzed during the current study are available from the corresponding author on reasonable request.

## Abbreviations

SSI: Surgical site infection
SWD: Surgical wound dehiscence
AKI: Acute kidney injury
PLIF: Posterior lumbar interbody fusion
BMI: Body mass index
CDC: Centers for Disease Control
SD: Standard deviation
IQR: Interquartile range
CNY: Chinese Yuan
OR: Odds ratio
CI: Confidence interval
